# Diagnostic and Treatment Difficulties of Pyelonephritis in Pregnancy in Resource-Limited Settings

**DOI:** 10.4269/ajtmh.2010.10-0332

**Published:** 2010-12-06

**Authors:** Rose McGready, Vanaporn Wuthiekanun, Elizabeth A. Ashley, Saw Oo Tan, Mupawjay Pimanpanarak, Samuel Jacher Viladpai-nguen, Wilarat Jesadapanpong, Stuart D. Blacksell, Stephane Proux, Nicholas P. Day, Pratap Singhasivanon, Nicholas J. White, François Nosten, Sharon J. Peacock

**Affiliations:** Shoklo Malaria Research Unit, Mae Sot, Tak, Thailand; Mahidol-Oxford Tropical Medicine Research Unit, Faculty of Tropical Medicine, Mahidol University, Bangkok, Thailand; Centre for Vaccinology and Tropical Medicine, Churchill Hospital, Oxford; Department of Microbiology, Imperial College National Health Service (NHS) Trust, London, United Kingdom; Department of Microbiology and Immunology, Faculty of Tropical Medicine, Mahidol University, Bangkok, Thailand; Department of Medicine, University of Cambridge, Addenbrooke's Hospital

## Abstract

Limited microbiology services impede adequate diagnosis and treatment of common infections such as pyelonephritis in resource-limited settings. Febrile pregnant women attending antenatal clinics at Shoklo Malaria Research Unit were offered urine dipstick, sediment microscopy, urine culture, and a 5-mL blood culture. The incidence of pyelonephritis was 11/1,000 deliveries (*N* = 53 in 4,819 pregnancies) between January 7, 2004 and May 17, 2006. Pyelonephritis accounted for 20.2% (41/203) of fever cases in pregnancy. *Escherichia coli* was the most commonly isolated pathogen: 87.5% (28/32) of organisms cultured. Susceptibility of *E. coli* to ampicillin (14%), cotrimoxazole (21%), and amoxicillin-clavulanic acid (48%) was very low. *E. coli* was susceptible to ceftriaxone and ciprofloxacin. The rate of extended spectrum β-lactamase (4.2%; 95% confidence interval = 0.7–19.5) was low. The rate and causes of pyelonephritis in pregnant refugee and migrant women were comparable with those described in developed countries. Diagnostic innovation in microbiology that permits affordable access is a high priority for resource-poor settings.

## Introduction

Although pyelonephritis affects only 1–2% of pregnant women, it is accompanied by significant maternal morbidity and fetal morbidity and mortality.^1^–^3^ Pyelonephritis can result in premature labor in 20–30% of women, and these infants are at high risk of neonatal death in resource-limited settings.^4^,^5^ Lack of access to microbiological facilities in these settings prevents antenatal care programs from providing the recommended^6^–^10^ routine urine culture to detect asymptomatic bacteriuria. This results in a failure to promptly diagnose urinary tract infection (UTI) and provide appropriate antimicrobial therapy.

Most resource-limited settings are also unlikely to have the information that they need to guide empiric antimicrobial therapy.^11^,^12^ Diagnosis and susceptibility profiles of urinary pathogens are hampered by numerous factors in populations such as displaced people living on the Thai–Burmese border. Difficulties in obtaining a clean urine sample, transportation limitations that can prevent timely arrival of specimens from the clinic to a microbiological facility, and use of shop-bought antibiotics or yaa-chud, which has been reported to contain antibiotics,^13^ could inhibit culture growth. In addition, there is a perceived lack of need on the part of the main health providers to collate such data for local populations.

Resource-limited settings are more likely to have access to microscopy than culture facilities. Reports on microscopy of the urine sediment in pregnancy refer mostly to its role in screening for asymptomatic bacteriuria.^14^ Boucher and others^14^ advocated that microscopy of urine sediment be stopped after urine culture was routine for screening at antenatal care clinics. One Turkish study found the sensitivity and specificity of microscopic urinalysis were 71.0% and 73.6%, respectively, in women with asymptomatic bacteriuria and symptomatic UTI, which was considerably higher than corresponding figures for dipstick testing of 38.7% and 35.8%, respectively; they elected to continue urine microscopy, because routine culture was not available.^15^ In a pregnant population in rural Australia, the positive predictive value of dipstick urinalysis in diagnosing asymptomatic bacteriuria was reported to be low (33.5%).^16^ Microscopy of urine sediment was introduced by Médecins sans Frontières in laboratories in refugee camps on the Thai–Burmese border, but the sensitivity and specificity for diagnosis of UTI has never been formally tested.

Although 10–14 days therapy is accepted for treatment of pyelonephritis,^17^ particularly in pregnant women, new studies are challenging the duration of therapy.^18^ The treatment choice for pyelonephritis in pregnant women is limited. Antimicrobial drug resistance is rising at an alarming rate, with very few new treatment options for Gram-negative bacteria in non-pregnant and pregnant individuals.^19^ The rise of extended spectrum β-lactamase (ESBL)-producing bacteria is compounding the problem, because antimicrobials such as the cephalosporins, which have a good safety profile in pregnant women, are rendered ineffective. Only four randomized control trials in pregnant women including 90,^20^ 178,^21^ 179,^22^ and 101,^23^ or 548 women in total, have assessed antimicrobial efficacy. These studies concluded that in non-bacteremic patients, oral cephalexin (500 mg every 6 hours) did not differ in efficacy and safety from intravenous (IV) cephalothin (1 g every 6 hours) treatment^20^; one time daily IV ceftriaxone was as effective as multiple daily doses of cefazolin.^22^ No difference in clinical response was observed with IV ampicillin and gentamicin, IV cefazolin, or intramuscular ceftriaxone,^23^ whereas cefuroxime (750 mg every 8 hours IV) was more efficacious and better tolerated than cephradine (1 g every 6 hours IV).^21^ A review article reported that 2 weeks of therapy seems acceptable for treatment of acute pyelonephritis in women, not specifically pregnant women^24^; nevertheless, 10- to 14-day courses are suggested.^25^,^26^

Here, we report the incidence, risk factors, diagnosis, microbial pathogens, antimicrobial susceptibilities, and outcome of pregnancies associated with pyelonephritis in antenatal clinics attended by refugees and migrants on the Thai–Burmese border. This aims to provide information to guide future empiric prescribing in this population, stimulate interest in surveillance work by non-government organizations (NGOs) responsible for the health of populations, and highlight the need for field-adapted susceptibility testing.

## Materials and Methods

The 41 pregnant women included were also included in a manuscript on the causes of fever in pregnancy published elsewhere.^27^

### Study sites and population.

At the time of this study, the Shoklo Malaria Research Unit conducted antenatal clinics on a weekly basis in Tak province, Northwest Thailand for approximately 800 pregnant Karen and Burmese women in Maela Refugee Camp and 300 migrant pregnant women in four villages (Maw Ker Thai, Murunchai, Walley, and later, Wang Pha). This is an area endemic for *Plasmodium falciparum* and *P. vivax* malaria^28^ with a low seroprevalence of human immunodeficiency virus (HIV) infection (< 0.5%).^29^

**Figure 1.**
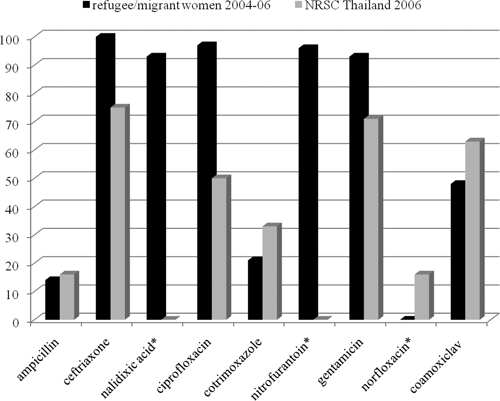
Antimicrobial sensitivity (%) in *E. coli* isolates in febrile pregnant migrant and refugee women (January 4 to May 6) and in outpatients from the National Resistance Surveillance Center, Thailand (2006). *Zero value means no data were available.

Before this study, antenatal screening for asymptomatic bacteriuria was done at 28 weeks gestation, which coincides with fetal viability in this setting. Pregnant women had mid-stream specimens of urine screened for blood and protein by dipstick (ROCHE Combur7 test D), and if the result was positive, the sample was centrifuged, and the sediment was examined by microscopy. No routine microbiology was available before this study.

### Inclusion and exclusion criteria.

We included any pregnant woman with confirmed fever (aural temperature > 37.5°C) who could give written informed consent and was able to follow a supervised treatment regimen. There were no exclusion criteria. Febrile women who did not want to participate in the study were provided with the same level of care as study participants. The consent form and information sheet for the study were available in Karen and Burmese languages. A trained study midwife read the forms to women who were unable to read.

### Laboratory procedures and sample collection.

After clinical examination, each pregnant woman had a standardized set of laboratory investigations to maximize the prospect of positively identifying the pathogen(s) causing her fever and to study comorbidities. The investigations reported here include urine dipstick, urine sediment microscopy, urine culture from a clean mid-stream urine sample, a 5-mL blood culture collected into a BacT/ALERT FA aerobic blood culture bottle, and complete blood count results. Midwives were trained in the specific instructions required for the urine sample collection. Instructions were given to each woman by the midwife. The cup with the clean catch urine was brought directly back to the midwives and sent to the field laboratory for processing. Samples for stick testing, microscopy, and culture were all prepared from the same urine specimen within 5 minutes of arrival to the laboratory. The urine stick and microscopy could be read on site, but the culture specimen was placed into a 10-mL plain sterile tube and maintained in the fridge for a few hours before being transported in an icebox to the microbiology laboratory in Mae Sot. Blood culture bottles were handled at room temperature until they reached the microbiology laboratory. There was no routine transport to the microbiology laboratory on Sunday, and samples collected before transport on Monday were stored at 4°C (urine) or room ambient temperature (blood culture; ambient temperature is approximately 30°C).

### Urine stick and sediment microscopy.

The urine dipstick (ROCHE Combur-10-test UV/M) was completely immersed in the urine, placed flat on a bench top for 60 seconds, and then read by the study technician. The test was considered positive if both nitrites and leukocytes were positive. A volume of 10 mL of fresh urine was centrifuged at 800 × *g* for 5 minutes. One drop of sediment was placed on a slide, and a coverslip was applied. The urine was examined with light microscopy using objective × 40 for 10 fields for each of the following: white blood cells (WBC), red blood cells (RBC), epithelial cells, crystals (cystine, oxalate, and phosphate), casts (granular, RBC, WBC, and hyaline), bacteria, yeast, and *Trichomonas*. The total count was divided by 10 to obtain the average per HPF. The minimum and maximum count from the 10 fields was also reported. Microscopy of urine sediment was considered contaminated when it contained ≥ 5 epithelial cells/high powered field (HPF; minimum value of the range). Sediment was considered positive when epithelial cells were < 5/HPF and there were bacteria (≥ 1+) and WBC (≥ 10/HPF; minimum value of the range) identified in the sediment. In centrifuged urine, 1+ = 1–10 bacteria/HPF, 2+ = 11–100 bacteria/HPF, 3+ ≥ 100 bacteria/HPF, and 4+ = field packed with organisms.^30^

### Bacteriological culture.

Quantitative urine culture was performed by using a sterile loop to inoculate 0.001 mL urine onto a 5% sheep blood agar and a MacConkey agar plate (Oxoid, Basingstoke, Hampshire, United Kingdom). All plates were incubated in air at 35°C and read at 24 and 48 hours for bacterial identification and colony count. Isolates from specimens containing a single bacterial colony type at greater than or equal to 10^5^ colony-forming unit (cfu)/mL were identified, and antimicrobial susceptibility tests were performed. The relevance of urine containing a single colony type between 10^4^ and 10^5^ cfu/mL was determined based on clinical features. Cultures were considered contaminated if more than one organism or non-pathogen were isolated. Cases with contaminated cultures were removed from further analysis. Blood samples were inoculated aseptically into blood culture BacT/ALERT FA bottles (BioMérieux, Durham, NC) containing 30 mL of media, activated charcoal, and an internal sensor that detects carbon dioxide as an indicator of microbial growth. Blood culture bottles were vented (using BCB Vent/Sub units; Difco, West Molesey, Surrey, United Kingdom) immediately and incubated unshaken in air for 7 days at 37°C. Gram stains were performed on smears prepared at 12–24 hours and 36–48 hours or when the sensor indicated growth. Bottles were subcultured routinely onto blood agar at 12–24 hours and 36–48 hours and after 7 days of incubation, and they were subcultured on days 3–6 if there was any suggestion of visible growth (that is, indicator changed). Antimicrobial susceptibility (including the detection of ESBL activity) was determined using disk diffusion methodology in accordance with Clinical and Laboratory Standards Institute guidelines.^31^

### Clinical procedures and definitions.

Women who consented to the study were given a study code, and a case record form was completed by the doctor on duty. All women were admitted to hospital, had their medical and obstetric history reviewed, and were given a full examination by a physician and a midwife. Patients' symptoms were defined and recorded using a standardized checklist. Treatment was initiated according to the results of the clinical examination and initial infective screen, and they could be changed if new results became available. Aural temperature, blood pressure, pulse, and respiratory rate were recorded every 6 hours. Patients were reviewed daily, and the symptom checklist was repeated. Body temperature was measured by an aural thermometer with disposable covers (Genius). Fever clearance time (FCT) was defined as the time from onset of treatment to the first time that aural temperature dropped below 37.5°C and remained < 37.5°C for 24 hours. Pyelonephritis was diagnosed if a significant growth on urine culture was accompanied by consistent clinical signs (systemic features such as fever, chills, nausea, vomiting, or flank pain or costovertebral angle tenderness) or if there were consistent clinical signs and a valid reason for the urine culture to be negative (e.g., antibiotics in the past 72 hours). Asymptomatic UTI was defined as a significant growth on urine culture and non-clinical symptoms or signs of UTI. Anemia was defined by a hematocrit < 30%, and severe anemia was defined by a hematocrit < 20%. Teenager was defined as aged < 20 years.

### Mother and infant follow-up.

After discharge, patients were followed up weekly at their regular antenatal clinic, where they were asked about fever and other symptoms and signs in the preceding week. Standardized symptom screening and physical and obstetric examinations were continued for 6 weeks. Women who had been diagnosed with pyelonephritis were reviewed weekly for 6 weeks with repeat urine dipsticks, repeat urine sediment, and cultures at days 14 and 42. Any positive sample by dipstick (WBC and nitrite positive) was followed by microscopy of urine sediment and culture. Any patient with symptoms at any time by day 42 had repeat urine stick, sediment, and culture. Deliveries were supervised whenever possible, although many women chose to deliver at home. Gestational age was assessed by ultrasound (≤ 24 weeks gestation) or by the Dubowitz score in women with either a late or no ultrasound.^32^ Birth weight was assessed in all infants that were seen after birth, but analysis for birth outcomes was restricted to those weighed in the first 72 hours of life. Where possible, all infants were examined again at 1 month of age. Low birth weight was defined by a birth weight < 2,500 g, pre-term delivery was defined by a gestational age < 37 weeks, and an abortion was defined by a delivery < 28 weeks.

### Treatment.

Paracetamol was given at a dose of 1 g every 8 hours for the first 48 hours if fever continued. Tepid sponging and fanning was used when necessary to control the fever. Patients were asked to drink at least 1 L of water every 8 hours or were treated with IV fluids if unable to tolerate oral fluids. Pyelonephritis was treated with ceftriaxone 1 g IV every 24 hours for 10–14 days unless susceptibility testing indicated a change to a different antibiotic.

### Analysis.

Data were described using the statistical program SPSS for Windows (Version 14; SPSS Benelux Inc., Gorinchem, The Netherlands). Continuous normally distributed data were described by the mean (standard deviation and range), and non-normally distributed data were described by the median (range). Percentages were given for categorical data. The sensitivity, specificity, and positive and negative predictive values of urine dipstick and urine sediment microscopy were compared with urine culture. Contaminated culture and urine sediment microscopy results were excluded for the purpose of this analysis.

### Ethical clearance.

Informed consent was obtained from all participants, and the study was approved by the Oxford Tropical Research Ethics Committee, United Kingdom (#013-03).

## Results

During the study period between January 7, 2004 and May 17, 2006, 203 febrile women consented to participate, of whom 41 were diagnosed with pyelonephritis and 1 was diagnosed with an asymptomatic UTI in association with acute *P. falciparum* infection. The mean maternal age of women with pyelonephritis was 26 ± 7 (16–40) years, of whom 39% (16/41) were primigravida and 19.5% (8/41) were teenagers. A total of 4,819 deliveries at Shoklo Malaria Research Unit (SMRU) were recorded during the same period. There were 12 further cases of pyelonephritis in pregnant women who did not participate in the study, giving an overall incidence of 1.1% (53/4,819) or 11/1,000 deliveries.

### Urine dipstick, urine sediment microscopy, and urine culture results.

Results were not available for urine dipstick in 3.4% (7/203) of women, urine sediment microscopy in 1.5% (3/203) of women, and urine culture in 0.5% (1/203) of women. The overall proportion of women with positive results on urine dipstick was high (Table 1). There were 82 of 196 (41.8%) women with detectable hematuria, and 38.8% (76/196) of women had ketones. There were 13.3% (26/196) positive for leukocytes and nitrite on dipstick testing, and 5.1% (10/196) were negative for leukocytes but positive for nitrite. Of the women whose urine samples tested positive for leukocytes and nitrites, 53.8% (14/26) had positive urine culture (Table 1).

There were 24 of 200 (12.0%) contaminated urine sediment samples using the criteria outlined above. After excluding these samples, positive urine sediment microscopy was observed in 28.4% (50/176) of samples. Of these, 54.0% (27/50) were urine culture positive, and in the women for whom dipstick was available, leukocytes and nitrites were positive in 30.6% (15/49) of samples. *Trichomonas* was detected in urine in 1% (2/200) of women.

In specimens where the urine sediment and urine culture were not reported as contaminated, urine culture results were reported as no or non-significant growth in 81.2% (138/170) and positive growth in 18.8% (32/170) of the samples. Of the 32 positive urine cultures, only 1 was considered to be unrelated to the presenting fever in a woman diagnosed and treated with artesunate for *P. falciparum* infection. Her fever resolved before the urine culture result (*Escherichia coli* > 10^5^ cfu/mL) was available, but she was treated with nitrofurantoin for 7 days and was culture negative on day 42 of follow-up.

In specimens where the urine sediment and urine culture were not reported as contaminated, urine sediment microscopy and urine dipstick results were compared with urine culture results using sensitivity, specificity, and positive and negative predictive values (Table 2). The use of blood and/or protein on urine dipsticks was poorly predictive for a positive urine culture. Whereas the combination of leukocytes and nitrites gave the highest positive predictive value for a positive urine culture, the sensitivity was only 44%, lower than nitrites alone (Table 2). The use of microscopy of urine sediment, which combines WBC, bacteria, and epithelial cells, provided the highest sensitivity, specificity, and negative predictive value overall, but the positive predictive value was low. In this series, lowering the urine sediment cut-off to WBC ≥ 7/HPF resulted in a higher sensitivity than WBC ≥ 10/HPF (Table 2), but it did not improve the positive predictive value.

### Pyelonephritis.

Of the 203 women in the original cohort, 41 women were diagnosed with pyelonephritis, of which 78.0% (32) had a positive urine culture. The diagnosis of pyelonephritis included 21.9% (9/41) of women with no significant growth on urine culture. Seven of these women had documented antibiotic intake in the 72 hours before the sample for culture. Urinary pathogens cultured from urine were *E. coli* (87.5%; 28/32), *Citrobacter* sp. (1/32), *Enterococcus* sp. (1/32), *Klebsiella* sp. (1/32), and *K. oxytoca*. Five of the specimens had a count of 10^4^ cfu/mL (three *E. coli* and one each of *Enterococcus* sp. and *K. oxytoca*), whereas the remainder contained at least 10^5^ cfu/mL.

All women with a positive urine culture had a hemoculture performed. One woman had a blood culture positive for a coagulase-negative *Staphylococcus*, which was presumed to be a contaminant, and of the remaining women, 25% (8/32) had concurrent positive hemoculture with the same organism (all *E. coli*).

### Antimicrobial susceptibility.

*E. coli* isolates were susceptible to ceftriaxone (100%), nitrofurantoin (96%), ciprofloxacin (97%), nalidixic acid (93%), and gentamicin (93%) (Figure 1). The rate of *E. coli* susceptibility to ampicillin (14%) and cotrimoxazole (21%) was very low. Susceptibility to amoxicillin-clavulanic acid (48%) was also low. Organisms isolated from hemoculture showed the same antimicrobial susceptibility patterns as the matched urinary isolate from the same patient. Susceptibility to ceftazidime and cefoxitin was reported for 24 of 29 *E. coli* isolates. Only one isolate was identified as producing an ESBL (4.2%; 95% confidence interval [CI] = 0.7–19.5). The single *Enterococcus* sp. was sensitive to ampicillin. The *Citrobacter* sp. was resistant to ampicillin and cotrimoxazole but sensitive to nitrofurantoin, ciprofloxacin, cephalosporins, and amoxicillin-clavulanic acid.

The antimicrobial susceptibilities of urinary pathogens on the Thai–Burmese border in refugee and migrant women were compared with data for *E. coli* isolates from outpatient UTI samples obtained from the National Resistance Surveillance Center (NRSC) of Thailand for 2006 (Figure 1). *E. coli* isolates from refugee and migrant women were more likely to be resistant to ampicillin/amoxicillin and cotrimoxazole than the *E. coli* in the 2006 NRSC Thailand community acquired series. Although rarely prescribed, amoxicillin-clavulanic acid had a lower rate of susceptibility for *E. coli* from refugee and migrant women.

### Risk factors for pyelonephritis.

The proportion of primigravida with pyelonephritis was significantly higher than multigravida (1.9% [21/1,082] versus 0.9% [32/3,737], *P* = 0.004). There was no significant difference in mean maternal age in women with (26.1 ± 6.7 years) and without pyelonephritis (26.7 ± 6.7 years, *P* = 0.531). The mean gestation at the time of infection was 22.8 ± 9.5 (5.6–41.2) weeks, with the highest frequency of infection in the second (51.2%; 21/41) followed by the third (31.7%; 13/41) and first trimesters (17.1%; 7/41). The mean gestational age of women at the time of diagnosis was significantly higher in women with bacteremia than those without bacteremia (30.7 ± 7.1 versus 20.4 ± 8.7 weeks, *P* = 0.004). Women with pyelonephritis associated with bacteremia (*N* = 8) had higher admission temperature, longer fever clearance times, lower blood pressure, and younger maternal age than those without bacteremia, although these were not significant (data not shown). No significant differences in hematological parameters at baseline were observed between women with and without bacteremia (data not shown). Ultrasound findings were not included here, because it was not consistently available for all women.

### Clinical symptoms and antimicrobial therapy.

**Figure 2.**
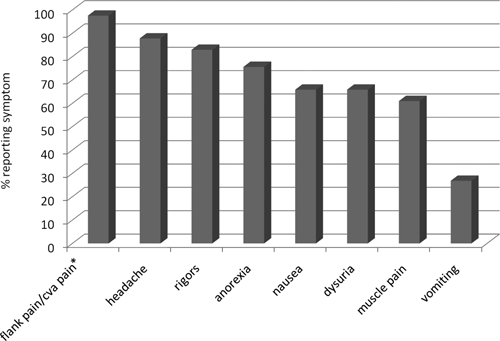
Symptoms reported on admission in pregnant women with pyelonephritis. Cva = costovertebral angle.

Women with pyelonephritis were very symptomatic (Figure 2). Costovertebral angle pain was the most common symptom reported. More than one-quarter of women reported vomiting. The median days of fever reported before diagnosis were 3 (1–10), with nearly one-half (46.3%; 19/41) presenting to the clinic with a history of fever of 2 days or less. Nearly one-half (48.8%; 20/41) of women reported antibiotic use at some stage in their pregnancy before admission, and 41.5% (17/41) of women reported prior treatment in their lives for UTI. There were 27 of 41 (65.9%) women who had renal tract ultrasound during their hospitalization. In one-half of these cases (51.9%; 14/27), the kidney was reported to be abnormal: nephrolithiasis in 3 women and hydronephrosis in 11 women.

Nearly all women (90.2%; 37/41) with pyelonephritis were treated with ceftriaxone 1 g daily in a single dose for 7–14 days. Three of the remaining women were treated with IV ampicillin plus gentamicin or oral amoxicillin-clavulanic acid, and one woman received azithromycin and nitrofurantoin. The median (range) time in days to fever clearance from admission was 2 (1–8). There were three women who had a culture-confirmed repeat episode of UTI after ceftriaxone treatment, two on day 42 with repeat *E. coli* and one on day 107 that was also *E. coli* after a first infection with *K. oxytoca.* The original and subsequent antibiograms of these three subsequent infections did not indicate more resistant strains. The day 42 cure rate of *E. coli* pyelonephritis treated with ceftriaxone in pregnancy was 91.9% (95% CI = 78.7–97.2).

### Pregnancy outcome.

Of the 41 women with pyelonephritis, 7.3% (3) left the study area before pregnancy outcome was known. Among the remaining 38 women, 94.7% (36) delivered (all live born) and 5.3% (2) aborted. The mean (± standard deviation) gestation of delivered infants was 38.9 ± 2.5 (29.2–42.5) weeks, and 8.3% (3/36) of infants were born pre-term (29 + 2, 32 + 0, and 35 + 4 weeks). Two of these premature births occurred while the woman was still on treatment: day 4 and day 7. Among women who delivered, 97.2% (35/36) of infants were weighed within 72 hours of birth. There were 14.3% (5/35) of infants with low birth weight, and the mean (± standard deviation) birth weight was 2,956 ± 573 g. There was one abnormal infant with Down's syndrome (gestation of UTI was 25.4 weeks). All infants, including the infant born at 29 weeks gestation, were alive at 1 month.

## Discussion

As expected, *E. coli* was the most commonly isolated urinary pathogen, accounting for 87.5% of acute pyelonephritis and confirming similar rates from previous reports in pregnancy.^17^,^20^,^33^–^36^ Ampicillin, previously the drug of choice for *E. coli* antepartum pyelonephritis because of its efficacy, cost, and safety for the mother and fetus, has been lost to global resistance.^37^ With a rate of ampicillin susceptibility for *E. coli* of only 14% on the Thai–Burmese border, it represents an unacceptable choice for febrile UTI in pregnancy. Low rates of susceptibility to ampicillin, cotrimoxazole, and amoxicillin-clavulanic acid have been reported in most of Europe^17^,^38^–^40^ and the United States.^37^,^41^ The low rates of susceptibility to ampicillin, cotrimoxazole, and amoxicillin-clavulanic acid and the high rates of susceptibility to ceftriaxone, nitrofurantoin, ciprofloxacin, nalidixic acid, and gentamicin in our study are likely to have resulted from antimicrobial prescribing patterns in the camp, which have not changed for 20 years. The refugee camps on the Thai–Burma border have used oral ampicillin or amoxycillin for outpatient UTI and IV ampicillin and gentamicin for hospitalized patients since their inception in 1986. The latest guidelines contain changes in recommended regimens for urinary tract pathogens based primarily on the evidence presented here.

More than 10 years ago, Biedenbach and others^42^ reported high antimicrobial resistance in Thailand among commonly encountered pathogens and a rate of ESBL production of 15.7% for *E. coli* and 45.6% for *K. pneumoniae*.^42^ The EBSL rate among urinary *E. coli* in this series (2004–2006) was low at 4%. Plausible explanations for the low rate of ESBL in these isolates are that (1) literature reports are often biased to nosocomial infections and this is highly likely to select for a higher rate of ESBL-producing organisms, and (2) the response rate to the treatment (ceftriaxone 1 g IV once daily) used suggests that ESBL was not common.^43^ ESBL rates are likely to change in the coming years with the change in prescribing practices and the high rates of ESBL-producing strains in *E. coli* reported from intra-abdominal infections in the Asia–Pacific region and Thailand (42.2% and 50.8%, respectively) in 2007.^44^

Without an overview of the organisms and susceptibility patterns, it is very difficult to generate useful guidelines and reduce death from sepsis in resource-limited settings.^10^ Medical care in refugee settings is often the domain of NGOs. Practice guidelines tend to be generated from headquarters and may not be country (even continent)-specific. Most NGOs use restricted lists of essential drugs. Collating and analyzing local microbiological results, if there are any, is rarely part of an NGO's mandate. If individual patient data cannot be collected, routinely intermittent surveillance could prove useful. This could improve survival in sepsis in resource-limited settings.^45^

The incidence of acute antepartum pyelonephritis was 1.1%, which is similar to that reported with antepartum universal screening (1–2%).^3^,^17^ This is reassuring, because a high rate of chronic renal disease has been reported from Thailand.^46^ Pyelonephritis accounted for 20% of fever presentations in febrile pregnant women on the Thai–Burmese border. This is in contrast to a fever study in adults conducted 300 km south of the sites in this manuscript, where only 2.1% (13/613) of diagnoses among febrile adults (aged 20–87 years, 53.1% male) were pyelonephritis.^47^ Concomitant *E. coli* bacteremia and urinary sepsis in women in this prospective cohort were higher (25%) than similar prospective studies where rates of 14.4% (13/90)^20^ or 8.4% (15/179)^48^ were reported. This reaffirms the need for prompt and effective treatment of this condition in this setting.

Pyelonephritis is essentially a clinical diagnosis. The sensitivity and specificity of leukocyte esterase and nitrites in this cohort are in agreement with a meta-analysis that found sensitivity and specificity of nitrites were around 50% and 95%, whereas leukocyte esterase was highly variable, with a sensitivity of 50–60% and a lower specificity than nitrites.^49^ It has been suggested that nitrites alone indicate the need for further urine testing and could be used to initiate treatment,^50^ which would overtreat a large percentage of patients in this setting. Urine sediment microscopy proved more sensitive than urine dipsticks for diagnosis of pyelonephritis. A WBC count of ≥ 7/HPF in the presence of bacteria and with epithelial cell count < 5/HPF predicted a positive urine culture with the highest sensitivity, but this was not as specific as WBC count of ≥ 10/HPF or the presence of leukocytes and nitrites on urine dipstick. In areas where microscopy is practiced routinely to diagnose malaria and microbiological facilities are not available, the urine sediment is likely to provide a cheap alternative for urine examination. Cut-offs are best confirmed with microbiological support, but this could be done as a survey. Known limitations of the dipstick nitrite test in diagnosing bacteriuria include infection with non–nitrite-producing pathogens and insufficient time since the last void for nitrites to appear at detectable levels (ideally, at least 4 hours); in this setting, another limitation is possibly a lack of dietary nitrate.^51^

The women studied here were a select group presenting with fever. The results are likely to be different than if all pregnant women had been screened to detect asymptomatic bacteriuria.^52^ This study suggests that antibiotics that can be used to treat pyelonephritis in this area include ceftriaxone, ciprofloxacin, and gentamicin. A number of publications including a meta-analysis on first trimester exposures suggest that ciprofloxacin use is not associated with increased risk of adverse events in pregnancy. Data from pregnant patients with inflammatory bowel disease and tuberculosis also suggest that ciprofloxacin is safe.^53^,^54^ Ceftriaxone requires IV or intramuscularly treatment, whereas ciprofloxacin can be given orally; additionally, patients can ambulate and be treated as outpatients. A 7-day regimen of nitrofurantoin has recently been described as an adequate choice for asymptomatic bacteriuria in Southeast Asia,^55^ and this recommendation is consistent with the susceptibilities reported here. Treatment changes based on the findings of this cohort were implemented in 2007. Microbiological capacity in the area has improved, and all suspected UTIs in pregnancy are cultured. In glucose-6 phosphate dehydrogenase deficiency negative women and unless otherwise suggested by an antibiogram, treatment is with 7 days of nitrofurantoin for uncomplicated UTIs and 10–14 days oral ciprofloxacin or IV ceftriaxone (if oral ciprofloxacin is not tolerated) for complicated UTI (fever, upper urinary tract signs, pyelonephritis, or abnormal ultrasound findings). In resource-limited settings, cost is an important issue: a 14-day course of oral ciprofloxacin (500 mg twice daily) purchased in Thailand costs approximately 80 baht (US $3.50) versus 980 baht (US $30.25) for ceftriaxone (1 g once daily; exchange rate is 32.4 baht to US $1). Screening for asymptomatic bacteriuria remains the ideal for pregnant women and is currently under consideration.

There was difficulty in this population to obtain a clean catch urine specimen. Up to 12% of specimens had a high number of epithelial cells, suggesting suboptimal specimen collection. Although a clean voided urine specimen with prior cleansing of the urethra and perineum is standard practice,^56^ there is no consistent recommendation for how this should be done.^17^ Attention to this detail must be included in screening asymptomatic women.

The birth outcomes in women with treated pyelonephritis, particularly low birth weight and prematurity, were similar to those recently reported in women prospectively followed in an uncomplicated malaria treatment trial from the same area in the same years.^57^ Further comparison on a larger number of women with pyelonephritis is planned.

In conclusion, acute pyelonephritis in pregnant refugee and migrant women occurred at the expected rate, and the predominant pathogen in this area was *E. coli*. Ampicillin, cotrimoxazole, and amoxicillin-clavulanic acid are unsuitable antimicrobials for the treatment of *E. coli* pyelonephritis on the Thai–Burmese border. Ceftriaxone and ciprofloxacin are more likely to result in cure of the patient. Ciprofloxacin is not licensed for use in pregnancy; however, recent literature and cost make it a viable alternative in resource-poor settings. Urine sediment fared somewhat better than urine dipsticks for diagnosis of culture-positive urine, but the positive predictive value of both were poor. Innovations in microbiology diagnostics that permit access at a low cost are a high priority for resource-poor settings.

## Figures and Tables

**Table 1:** Urine dipsticks in febrile pregnant women on the Thai–Burmese border 2004–2006

Investigation	Result	*N* (196)	Percent
Leukocyte (alone)	Negative	98	50.0
	1+	37	18.9
	2+	36	18.4
	3+	25	12.8
Nitrites (alone)	Positive	36	18.4
	Negative	160	81.6
Leukocyte and nitrites	Leukocyte and nitrite negative	88	44.9
	Leukocyte and nitrite positive	26	13.3
	Leukocyte positive and nitrite negative	72	36.7
	Leukocyte negative and nitrite positive	10	5.1
Protein	Negative	110	56.1
	1+	76	38.8
	2+	8	4.1
	3+	2	1.0
Ketones	Negative	120	61.2
	1+	28	14.3
	2+	26	13.3
	3+	22	11.2
Glucose	Negative	189	96.4
	> 1+	7	3.6
Blood	Blood	82	41.8
	RBC	56	68.3 (56/82)
	Hemoglobin	26	31.7 (26/82)
	No blood	114	58.2

**Table 2:** Comparison of urine sediment microscopy and urine sticks as predictors of positive urine culture results

	Urine sediment microscopy		Urine stick leukocyte and nitrite			Urine stick blood and protein			
	Epithelial cells < 5; WBC ≥ 10	Epithelial cells < 5; WBC ≥ 7	Leukocyte and nitrite positive	Leukocyte positive	Nitrite positive	Blood	Protein	Blood and protein	Blood or protein
Sensitivity (%)	84	97	44	88	47	72	56	44	72
Specificity (%)	84	82	92	59	86	66	60	82	41
Positive predictive value (%)	55	55	58	34	46	33	25	37	22
Negative predictive value (%)	96	99	80	95	87	91	85	86	87

Epithelial cells < 5/HPF using lower limit of range.
